# Practical Approaches to High-Risk Anastomoses in Robotic Pancreatoduodenectomy

**DOI:** 10.1245/s10434-025-17402-w

**Published:** 2025-05-06

**Authors:** Francesca Marcucci, Giovanna Carillo, Patricia Sánchez-Velázquez, Alberto Garcia-Picazo, Fernando Burdio, Benedetto Ielpo

**Affiliations:** Hepato‑Biliary and Pancreatic Surgery Unit, Department of Surgery, Hospital del Mar, Pompeu Fabra University, Barcelona, Spain

**Keywords:** Pancreatoduodenectomy, Minimally invasive surgery, Pancreatic cancer, Pancreas, Pancreatic fistula

## Abstract

**Background:**

Pancreatoduodenectomy (PD) is performed for the treatment of pancreatic head and periampullary tumors and is associated with relatively high postoperative morbidity and mortality. Traditionally conducted as open surgery, PD has evolved with the advent of minimally invasive techniques, including robotic-assisted approaches.

**Results:**

As reported in the literature, minimally invasive PD is becoming a safe and effective alternative surgical approach. Clinically relevant postoperative pancreatic complications, such as fistulas and hemorrhage, remain among the most challenging issues after PD, particularly in high-risk cases. There are several maneuvers that may reduce their incidence and mitigate their postoperative clinical impact.

**Conclusions:**

In this video, we describe strategies implemented at our center for high-risk PD cases, including some key tips and tricks.

**Supplementary Information:**

The online version contains supplementary material available at 10.1245/s10434-025-17402-w.

Pancreatoduodenectomy (PD) is a complex surgical intervention performed for malignant or premalignant lesions in the periampullary region.^1^ This demanding surgery involves intricate resections and reconstructions, making it prone to complications such as hemorrhage and anastomotic leaks.

Robotic PD has emerged as an alternative approach, offering enhanced precision and control through minimally invasive techniques. However, one of the most important topics is the safer handling of challenging cases, such as those involving small bile ducts and high-risk pancreatic stumps, likely to have clinically relevant postoperative pancreatic and biliary fistula.^2^

This multimedia video article presents practical strategies and innovative techniques developed to address common challenges in robotic PD, including preventing pancreatic and biliary fistulas and managing complex anastomoses. These approaches aim to provide surgeons with actionable insights to improve safety and efficacy in high-risk robotic PD cases.

## Placement of Pigtail Drainage beneath the Pancreatic Anastomosis

The first step involves placing pigtail drainage posteriorly to the future pancreatic anastomosis. This is essential to prevent the dissemination of amylase into the peritoneum cavity. In minimally invasive techniques, this placement can be challenging and most of the time, the drainage is not correctly placed. As shown in the video, we recommend placing the drainage just before completing the pancreatic anastomosis.

**Fig. 1:**
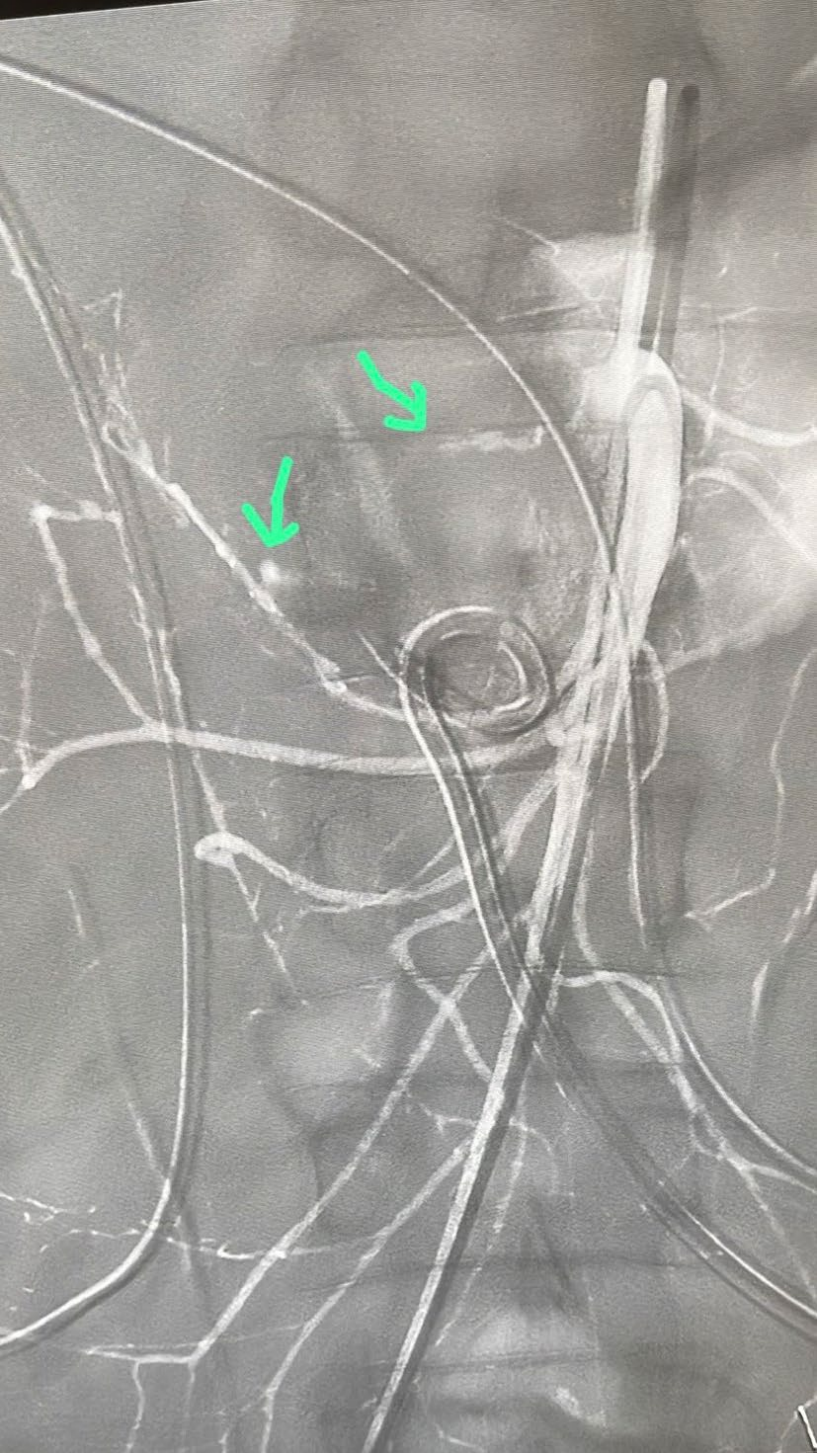
Pigtail catheter positioned near two bleeding points from branches of the superior mesenteric artery (green arrows)

The use of a pigtail drain offers several advantages. Firstly, its shape minimizes movement from the pancreatic area. Secondly, in cases of pancreatic fistula, it allows for repeated washing to reduce amylase concentration. Additionally, it can be repositioned under radiological guidance using a wire if necessary. Furthermore, it can be used to introduce hemostatic materials for bleeding control, as shown in Fig. 1. The image depicts a pigtail catheter positioned near two bleeding points from branches of the superior mesenteric artery, allowing for the injection of a hemostatic matrix. We recommend using a 12 Fr pigtail, which is sufficiently large to collect potential pancreatic fluid and inject hemostatic matrix.

Our practice is to perform a modified Blumgart anastomosis using two Goretex stitches, as demonstrated in the video. To minimize the opening of the jejunal loop and potentially reduce the incidence of pancreatic fistulas, we use a needle with coagulation. This tip is especially useful in case of small pancreatic duct, as in the case of the video. Adapting the size of the duct to the mucosa is an important strategy to minimize fistulas.

## Placement of a Biodegradable Stent in the Main Pancreatic Duct

To further reduce the risk of clinically relevant pancreatic fistulas, we place an intraductal stent before tightening the interrupted sutures of the duct to the mucosa.^3^ We use the ARCHIMEDES™ Biodegradable Pancreatic Stent (Q3 Medical), which features a dual drainage design with a helical outer and open inner lumen allowing pancreatic fluid flow through both the inside and the outside of the prosthesis while maintaining ductal patency and allowing for side branch flow.

Usually, we place a medium-degrading stent (approximately 20 days for degradation) with a size of 6 Fr and a length of 80 mm. After stent placement, we proceed with the anterior layer of the anastomosis using interrupted 5/0 Monocryl sutures, as with the posterior layer. Once the duct-to-mucosa anastomosis is completed, the Goretex reinforcement sutures are applied at the corners to strengthen these vulnerable areas. Finally, the transpancreatic U sutures are progressively tightened and secured, step by step, as shown in the video.

## Biliary Anastomosis

The biliary anastomosis consists of a double posterior layer secured with two continuous sutures. The first layer is performed on the posterior wall, incorporating the areolar tissue surrounding the bile duct.

This step is completed before opening the jejunal loop, as mucosal prolapse can impair visibility. The anastomosis is completed with a single continuous anterior suture. To facilitate the procedure, it is crucial to transect the bile duct at an oblique angle, ensuring the posterior wall of the bile duct is longer, which simplifies the anastomosis, as depicted in the video.

### V-Plasty of the Bile Duct to Enlarge Biliary Size

For cases involving very small bile ducts, as in the case included in the video, a V-plasty is an excellent strategy to enlarge the duct size.^4^ Using the robotic platform, a small axial incision is made at the anterior margin of the bile duct, and the corners are excised, as shown in the video. We use a 5/0 resorbable suture, which is easily handled by robotic drivers.

### Placement of Kehr Drainage in the Biliary Anastomosis

In high-risk cases, placing a T tube “Kehr” drain through the biliary anastomosis can be effective. This reduces bile flow through any potential pancreatic fistula and prevents mixed biliopancreatic fistulas. The drain remains open until pancreatic fistula resolution, typically confirmed within almost 5 days post-surgery. Afterward, it is closed and later removed, usually 2 months postoperatively. If a fistula persists, the drain remains open until resolved. This strategy helps minimize the impact of the bile liquid leaking from the jejunal duct to mucosa fistula.

#### Strategies to Prevent Post-Surgical Hemorrhage

##### Placement of a Biodegradable Collagen-Based Pad

In the event of a pancreatic fistula, hemorrhage is a common complication. To mitigate this risk, we place a biodegradable collagen-based patch over the superior mesenteric vessels. This is done prior to completing the pancreatic anastomosis. The patch is positioned and compressed for 2 min using dry gauze.

##### Wrapping the GDA Stump with the Teres Hepatis Ligament

Preventing bleeding from the gastroduodenal artery (GDA) stump is critical.^5^ As shown in the video, we wrap the GDA stump with the teres hepatis ligament. This involves dissecting the falciform ligament and securing the wrap with one or two sutures around the common hepatic artery and GDA.

## Conclusions

This video article highlights essential strategies for managing high-risk cases in robotic PD, offering practical solutions to address common challenges such as pancreatic fistulas, bile duct reconstruction, and hemorrhage prevention.

We briefly evaluated our experience in high-risk cases (7 to 10 according to Callery classification).^6^ Since incorporating these tips, the incidence of clinically relevant postoperative PD complications (grades B and C) has been reduced to approximately 20%.^7^ However, a larger multicenter prospective study should be conducted to further evaluate these findings.

By integrating these techniques into their practice, surgeons can enhance the safety and efficacy of this complex procedure. The detailed tips and use of robotic technology demonstrated in the video provide valuable insights, making this resource indispensable for surgeons adopting or refining their approach to robotic PD

## Electronic supplementary material

Below is the link to the electronic supplementary material.

Supplementary file 1 (MP4 79184 kb)
