# Correction: Practical Approaches to High-Risk Anastomoses in Robotic Pancreatoduodenectomy

**DOI:** 10.1245/s10434-025-17539-8

**Published:** 2025-05-14

**Authors:** Francesca Marcucci, Giovanna Carillo, Patricia Sánchez-Velázquez, Alberto Garcia-Picazo, Fernando Burdio, Benedetto Ielpo

**Affiliations:** Hepato‑Biliary and Pancreatic Surgery Unit, Department of Surgery, Hospital del Mar, Pompeu Fabra University, Barcelona, Spain

**Correction to: Ann Surg Oncol** 10.1245/s10434-025-17402-w

Giovanna Carillo's family name is correct as reflected here. The original article was corrected.

